# Postural stabilization and balance assessment in Charcot–Marie–Tooth 1A subjects

**DOI:** 10.1016/j.gaitpost.2014.07.006

**Published:** 2014-09

**Authors:** T. Lencioni, M. Rabuffetti, G. Piscosquito, D. Pareyson, A. Aiello, E. Di Sipio, L. Padua, F. Stra, M. Ferrarin

**Affiliations:** a Biomedical Technology Department, IRCCS Foundation Don Carlo Gnocchi Onlus, Milano, Italy; b Unit of Clinic of Central and Peripheral Degenerative Neuropathies, IRCCS Foundation, C. Besta Neurological Institute, Milano, Italy; c Department of Neuroscience, Ophthalmology and Genetics, University of Genoa, Genova, Italy; d Centro S. Maria della Pace, Foundation Don Gnocchi Onlus, Roma, Italy; e Polo Riabilitativo del Levante Ligure, Foundation Don Gnocchi Onlus, Sarzana, Italy

**Keywords:** Charcot–Marie–Tooth, Balance, Posture, Muscle strength, Plantar flexion

## Abstract

The aim of the present study was to assess postural stabilization skill in adult subjects affected by Charcot–Marie–Tooth disease (CMT) type 1A. For this purpose ground reaction force (GRF) was measured by means of a piezoelectric force platform during the sit-to-stand (STS) movement, until a steady state erect posture was achieved. Specific indexes to quantify Centre of Mass acceleration, both during postural stabilization and during quiet standing, were computed using a mathematical model. Forty-seven CMT1A subjects were recruited for the study, and the control group was formed by forty-one age- and sex-matched healthy subjects.

The results show that CMT1A subjects are less stable than controls during the quiet stance. Greater difficulty (high values of *Y*_inf_, the final instability rate) to maintain erect posture appears to be mainly associated with plantar-flexor muscle weakness, rather than to damage of the proprioceptive system. The worst performances shown by CMT1A subjects in the stabilization phase (high values of *I*, the global index of postural stabilization performance) seem to be associated with reduced muscle strength and the loss of large sensory nerve fibres.

Distal muscle weakness appears to affect both postural stabilization and quiet erect posture. The presented protocol and the analysis of postural stabilization parameters provide useful information on CMT1A balance disorders.

## 1 Introduction

Charcot–Marie–Tooth disease (CMT) is the most common inherited neuropathy, and the most frequent demyelinating subtype is CMT1A, representing 40–50% of all CMT cases. The typical CMT1A phenotype is characterized by symmetrical, and slowly progressive, distal muscle weakness and wasting, sensory impairment, foot deformities, and absent or reduced deep tendon reflexes [1]. As CMT influences daily life activities involving gait and balance [2], patients’ Quality of Life is affected [3]. Various studies have addressed the assessment of motor function in CMT subjects, focusing especially on gait analysis [4], [5], [6], the monitoring of gait pattern changes (in a longitudinal study) [7], and challenging locomotor tasks [8]. Although balance impairment is a critical aspect, and rehabilitation protocols have been proposed to improve the balance of CMT subjects [9], few studies have addressed balance impairment by a quantitative approach [10]. Different factors contribute in CMT imbalance (e.g. skeletal deformities and pain) [9], but a major challenge to maintaining postural control has been attributed to the progressive loss of proprioception [10].

In order to stay in, or to reach, an upright position, it is essential to have correct functioning of motor control. The muscle spindles send information to the nervous system concerning muscle contraction speed, through large sensory fibres, and related to muscle length, through smaller sensory fibres. Indeed, Dyck et al. found that reduced vibration sense was related to the selective disappearance of larger sensory fibres [11]. The same authors showed that CMT1A patients suffer from the loss of large sensory fibres, whereas smaller fibres are less affected [12].

Using standard posturographic analysis, Nardone et al. [13] found a significant increase in the sway area of the Centre of Pressure (CoP) in CMT1A subjects, compared to healthy subjects. Following on from this finding, the authors divided CMT1A subjects into two subgroups according to their Neuropathy Score [14], and found that the sway area of the more severely affected subjects was significantly larger than in the control group. Conversely, the sway of the less severely affected patients was no different from the controls. The lower motor conduction velocity found in the more severely affected group suggested a relevant involvement of smaller sensory fibres. This led Nardone et al. to hypothesize that, in CMT1A patients, large sensory fibre degeneration does not necessarily result in an altered steady erect posture as the smaller fibres possibly play a primary role and might be enough to maintain postural control [15], [16]. Instead Van der Linden et al. [17] argued that a deficit in large sensory fibres could contribute to postural stability impairment; their reasoning was that in CMT1A subjects they had found a correlation between vibration sense (a measure of large sensory afferent function) and the root mean square (RMS) values of CoP velocity, an index more sensitive to high frequencies than to body sway. There is still controversy concerning the influence of the somatosensory system on balance in CMT1A subjects.

Large sensory fibres are activated by moving dynamic stimuli, and it was for this reason that Nardone et al. [15] hypothesized that the significant involvement of such fibres would lead to greater postural performance impairment in dynamic postural tasks rather than in steady ones. Their hypothesis was only partially confirmed since the authors found that the CMT1A patients were only slightly worse than the controls in balancing on an actively moving dynamometric platform.

From the biomechanical point of view, human quiet standing can be described as the motion of an inverted pendulum pivoting around the ankle joints [18]. As a consequence, the muscles responsible for foot dorsi- and plantar-flexion play an important role in maintaining an erect posture. Therefore it has been suggested that postural stability in CMT1A subjects might be partly related to lower limb muscle strength [17], although such an association has not yet been established [13], [17].

In patients with CMT, more demanding tasks such as toe-walking and heel-walking were found to evidence locomotor deficits better than did natural walking [7]. Similarly, in balance studies, we expect the sit-to-stand task to be more demanding and, therefore, more sensitive to balance disorders than the quiet erect posture. In a previous study we developed a method, based on dynamometric platform data, to carry out both static and dynamic analysis of subject performance during a sit-to-stand task, focusing on “postural stabilization” [19]. This was defined as the condition where the set of processes of motor control and muscle activation no longer produce macroscopic motion, thus leading to the steady-state erect posture [19]. As we have already demonstrated in a group of healthy individuals, postural stabilization is more challenging than quiet erect posture because, before reaching final equilibrium, the subjects must dissipate any residual energy associated with body motion [19]. In subjects with motor control impairment, such as subjects suffering from CMT1A disease, an assessment of postural stabilization ability can allow the evaluation of the effects of sensory input and muscle strength on two different postural conditions, dynamic and static.

The goal of the present study was to apply the postural stabilization paradigm to a group of CMT1A patients and to a healthy control group. The aim was to investigate whether CMT1A patients show altered postural stability skills during quiet standing and/or during the postural stabilization phase, when compared to controls. If any such difference was evidenced, we wanted to analyze the possible role of reduced muscle strength and sensory large fibre loss, characteristics of CMT1A disease, in both quiet standing and during the postural stabilization phase.

## 2 Materials and methods

### 2.1 Subjects and clinical evaluation

Forty-seven Charcot–Marie–Tooth 1A adult subjects (29 females) with a wide range of severity levels were recruited for the present study. Forty-one age- and sex-matched healthy subjects formed the control group (21 females).

All the CMT1A subjects underwent a clinical assessment: Charcot–Marie–Tooth Examination Score (CMTES; ranging from 0, normal, to 28, worst [20]), Walk12 (score ranging from 0, no limitation, to 60, severe limitation [21]) and Overall Neuropathy Limitations Scale (ONLS, peripheral neuropathy disability scale, ranging from 0, no limitation, to 12, severe disability [22]). In detail, CMTES is calculated as the sum of the symptoms (sensory symptoms, motor symptoms legs, motor symptoms arms) and the signs (pin sensibility, vibration sense, strength legs and strength arms). Scoring for CMTES items range from 0 (no deficit) to 4 (severe impairment). ONLS is the sum of the leg score (ONLS_leg_, ranging from 0 when walking is not affected, to 7, if patient is restricted to wheelchair or bed) and arm score (ONLS_arm_, ranging from 0, no impairment, to 5 if all purposeful arm movements are prevented). For each CMT1A subject the Visual Analogue Scale (VAS) pain score was also acquired (0 no pain, 10 most severe pain) [23]. Ankle plantar-flexor (APF), dorsi-flexor (ADF), hip flexor, knee flexor and knee extensor muscles strength was assessed according to MRC scale (0, no movement 5 full strength) [24]. No patient presented relevant foot deformities.

Table 1 shows the relevant demographic and anthropometric data for the control and CMT1A groups, and clinical data for the CMT1A group.

**Table 1:** Demographic and anthropometric data for control and CMT1A group and clinical data for CMT1A group; mean (SD).

	Group	Controls	CMT 1A
	*N*	41	47
	Age [years]	44.1 (18.1)	44.5 (12.0)
	Height [cm]	169.0 (10.7)	166.8 (11.1)
	Body mass [kg]	68.2 (14.5)	67.1 (15.3)
	Total	–	7.6 (3.8)
	Sensory symptoms	–	0.9 (1.1)
	Motor symptoms legs	–	1.1 (0.6)
CMTES	Motor symptoms arms	–	0.6 (0.6)
	Pin sensibility	–	1.3 (1.0)
	Vibration sense	–	1.1 (0.8)
	Strength legs	–	1.4 (1.0)
	Strength arms	–	1.3 (1.0)
	Hip flexor	–	4.9 (0.2)
	Knee flexor	–	4.9 (0.2)
MRC	Knee extensor	–	4.8 (0.2)
	Ankle dorsi-flexor (ADF)	–	3.3 (1.5)
	Ankle plantar-flexor (APF)	–	4.2 (1.2)
	Total	–	3.0 (1.6)
ONLS	Legs	–	1.7 (0.9)
	Arms	–	1.3 (1.1)
VAS pain	Total	–	3.1 (2.8)
Walk12	Total	–	27.1 (10.7)

MRC, Medical Research Council scale for muscle strength; CMTES, Charcot–Marie–Tooth examination score; ONLS, Overall Neuropathy Limitations Scale; VAS, Visual Analogue Scale.

### 2.2 Protocol and data analysis

The protocol included a sit-to-stand (STS) task leading to erect posture on a piezoelectric force platform (Kistler, Switzerland, 960 Hz), in order to measure ground reaction force (GRF).

The STS movement was chosen because it is a functional daily task that has proved to be reliable, valid, sensitive, and predictive of falls, and of future locomotor and ADL status in frail subjects [25]. The STS task implies both upward and forward Centre of Mass (CoM) displacement, and the kinetic energy needs to be controlled after CoM has reached the final vertical position [19]. The STS task was performed as follows: the subjects were asked to move from the sitting position to a standing one (upright posture) at a self-selected natural speed, and, on standing, to stand as still as possible for at least 20s in an upright posture, looking at a target placed at eye level approximately 1 m away. The subjects stood up from a standard rigid chair (seat height: 43 cm [26]) without armrests, placed just outside the platform. The seated subjects were positioned with a knee angle of about 90°, feet parallel and laterally placed at a distance equal to the distance between the anterior–superior iliac spines. The subjects were then asked to perform the STS task with no assistance from the upper limbs and without moving the feet throughout the task. The task was repeated three times, a compromise between participant fatiguing and test reliability, as described in [19].

**Fig. 1:**
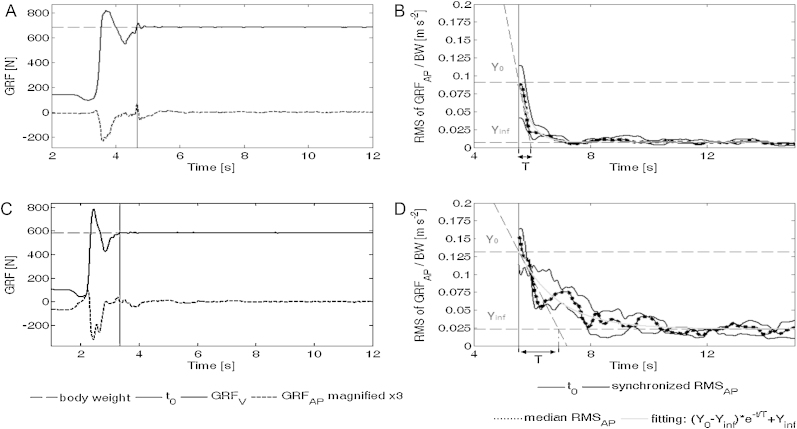
(A) and (C) Algorithm for *t*_0_ identification based on an analysis of vertical and anterior–posterior components of ground reaction force (GRF_V_ and GRF_AP_) derived by Etnyre et al. [26] for a healthy subject and a severe CMT1A patient. For better visualization, GRF_AP_ is magnified by a factor 3 identification of *t*_0_ (time instant corresponding to the end of macroscopic movement): (1) maximum vertical force peak is identified (corresponding to maximum vertical inertia); (2) following minimum force peak is identified (corresponding to minimum vertical inertia); (3) *t*_0_ is defined as the first sample higher than body weight. (B) and (D) Root mean square of antero-posterior component of the ground reaction force (RMS_AP_) and fitting of the negative exponential model plotted versus time for a healthy subject and a severe CMT1A subject with all model parameters altered.

The procedure for computation of the parameters is fully described in [19], and is summarized as follows. For each of the three trials, the time instant *t*_0_, corresponding to the end of macroscopic movement, was computed on the vertical component of GRF [26] as the first sample higher than body weight after the maximal force peak and the subsequent minimum force peak (Fig. 1 A and C). Then, for each trial, the root mean square of the antero-posterior component of GRF (RMS_AP_) was computed in 1s moving windows. After synchronization of the three RMC_AP_ profiles to the *t*_0_ instant, the median value for each time instant was then computed, providing a median profile. This last was finally fitted with a negative exponential model that allowed the identification of the independent parameters *Y*_0_ [m s^−2^], *T* [s] and *Y*_inf_ [m s^−2^] described below (Fig. 1B and D). Compared to other approaches for the analysis of postural stabilization, based on the sway of CoP, the negative exponential model method was found appropriate and more able to be generalized [27].

*Y*_0_ and *T* respectively quantify the instability rate at *t*_0_ and the time related to the stabilization phase duration (three times *T* is assumed as the time lag needed to reduce instability from *Y*_0_ to *Y*_inf_), while *Y*_inf_ quantifies the final asymptotic instability rate after achieving stabilization, and is comparable to classical posturographic sway indexes as it accounts for residual stationary postural oscillation.

It was shown [19] that healthy subjects are characterized by scattered *Y*_0_ and *T* values, but there is a limit value in their product, evidenced by a limiting hyperbolic curve in the *Y*_0_, *T* plane. This prompted the definition of another parameter *I* = *Y*_0_ * *T* [m s^−1^] that can be considered a comprehensive stabilization index.

Hereinafter, what happens in the time interval between *t*_0_ and *t*_0_ + 3*T* is defined as “postural stabilization”, while what happens after *t*_0_ + 3*T* is defined as “quiet standing”.

The study was approved by the local Ethical Committee, and all subjects signed informed consent forms.

### 2.3 Statistical analysis

Statistical analyses were performed using Matlab^®^ (MathWorks Inc., MA, USA). After verifying that the data were not normally distributed, all the analyses were conducted using non-parametric tests. The Mann–Whitney test was used to compare the data of the CMT1A group and the controls. Correlation analyses were performed between clinical scores and the two global performance parameters, *I* and *Y*_inf_, using the Spearman correlation test. *P*-values <0.05 were considered statistically significant. Correlation analysis was adjusted for multiple correlations according to the Holm–Bonferroni correction. To interpret the magnitude of the correlation coefficients *ρ*, the following guidelines from [28] were followed: for absolute values between 0 and 0.19 a very slight relationship, between 0.20 and 0.39 a slight one, between 0.40 and 0.59 moderate relationship, between 0.60 and 0.79 a strong one, and between 0.80 and 1 very strong.

## 3 Results

Table 2 shows the mean and standard deviation values of the biomechanical parameters computed from the postural stabilization task for controls and CMT1A patients.

**Table 2:** Postural stabilization parameters for control and CMT1A group; mean (SD).

Parameters	Controls	CMT 1A
*T* [s]	0.78 (0.40)	1.21 (0.58)**
*Y*_0_ [m s^−2^]	0.084 (0.038)	0.092 (0.039)
*I* [m s^−1^]	0.059 (0.024)	0.106 (0.072)**
*Y*_inf_ [m s^−2^]	0.010 (0.003)	0.018 (0.013)**

*T*: time duration of postural stabilization; *Y*_0_: residual instability at the beginning of the stabilization phase; *I*: global index of performance during stabilization; *Y*_inf_: the residual instability after stabilization in quiet standing.

^*^ Statistical significant differences between Controls and CMT1A are indicated with *p* < 0.05.

** Statistical significant differences between Controls and CMT1A are indicated with *p* < 0.001. ^***^ Statistical significant differences between Controls and CMT1A are indicated with *p* < 0.0001.

The CMT1A group had significantly higher values of *T*, *Y*_inf_ and *I* parameters with respect to the normal group, while between the groups *Y*_0_ was not significantly different.

**Fig. 2:**
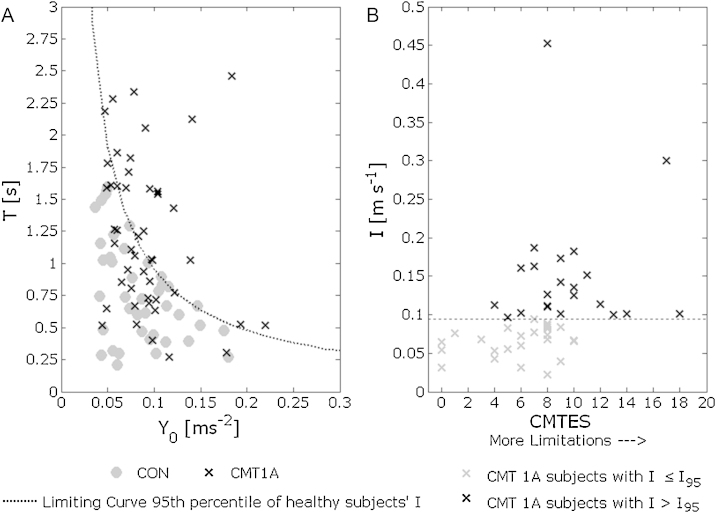
(A) Scatter-plots of *T* vs. *Y*_0_ for control and CMT1A groups during STS task. Dotted line represents the limiting curve of control group *Y*_0_ * *T* = *I*_95_, the 95th percentile of healthy subjects’ *I*. (B) Scatter plot of *I* vs. CMTES. In grey, subjects with parameter *I* lower than the limit of the 95th percentile of the controls distribution (dotted line represents *I*_95_ = 0.095 [m s^−1^]).

Fig. 2A shows the scatter-plots of *T* vs. *Y*_0_ for both the control and CMT1A groups; 55% of the CMT1A subjects fall within the limiting curve of the control subjects (95th percentile), while the remaining 45% presented higher *T* and *Y*_0_ values than healthy controls, migrating in the upper-right region of the plane: this phenomenon, interpretable as a condition of increased unbalance risk, is quantified by parameter *I*. This is consistent with the clinical status of patients. In fact, Fig. 2B shows that most of the mildly affected CMT1A patients (75% of those with a CMTES ≤ 6) had *I* ≤ *I*_95_ (the 95th percentile of the *I* values distribution of the healthy subjects), while most of the severely affected patients (58% of those with CMTES > 6) had an *I* > *I*_95_. In addition a significant correlation between the parameter *I* and the CMTES was found (*ρ* = 0.47, *p* < 0.001).

The association between clinical parameters and parameters related to the postural stabilization phase and quiet standing performances is reported in Table 3. Significant correlations with parameter *I* were found for the following factors: vibration sense and strength of dorsi- and plantar-flexors muscles. No influence of the proximal muscles of hip and knee joints (*p* > 0.05) on parameter *I* emerged, while parameter *Y*_inf_ correlated significantly with MRC_ADF_, Knee Flexor and Knee Extensor MRC. No correlation emerged between the parameter *Y*_inf_ and vibration sense or hip muscle strength. No relevant correlation with age and VAS pain was found for any biomechanical parameter.

**Table 3:** Correlation coefficient values *ρ* between postural stabilization parameters and CMTES, VAS pain, vibration sense, strength legs, distal and proximal muscles MRC.

Clinical parameters	*I* [m s^−1^]	*Y*_inf_ [m s^−2^]
CMTES total	0.47**	0.28
VAS pain	−0.03	0.07
Vibration sense	0.31*	0.01
Strength legs	0.57**	0.34*
MRC ankle dorsi-flexors (ADF)	−0.46**	−0.37*
MRC ankle plantar-flexors (APF)	−0.41*	−0.66***
MRC knee flexors	−0.12	−0.39*
MRC knee extensors	−0.13	−0.38*
MRC hip flexors	−0.16	−0.30

MRC, Medical Research Council scale for muscle strength; CMTES, Charcot–Marie–Tooth examination score.

* The level of statistical significance of *ρ* coefficients is indicated with *p* < 0.05.

** The level of statistical significance of *ρ* coefficients is indicated with *p* < 0.001.

*** The level of statistical significance of *ρ* coefficients is indicated with *p* < 0.0001.

## 4 Discussion

An analysis of postural stabilization after a STS task allowed us to study the postural behaviour of CMT1A subjects, in both dynamic conditions (characterized by *Y*_0_, *T* and *I*) and steady conditions (characterized by *Y*_inf_).

The CMT1A patients were less stable than the control group in quiet standing (higher values of *Y*_inf_) despite having preserved, or only slightly damaged, smaller sensory fibres sensitive to muscle length and most likely responsible for motor control in balance [13]. The correlation between *Y*_inf_ and leg strength score (a global measure of muscle weakness) seems to indicate that the difficulty of the CMT1A group in maintaining quiet standing is associated with muscle weakness, rather than with the proprioceptive loss of large sensory nerve fibres whose index (vibration sense) did not correlate with *Y*_inf_. The strong correlation between *Y*_inf_ and MRC_APF_ suggests that quiet standing relies more on plantar-flexor strength than on dorsi-flexors and/or proximal muscles. Our results support the clinical observations reported by Rossor et al. [29]; they found that CMT patients with significant distal weakness were more unsteady than CMT patients without, or with only mild signs of, muscle weakness, and argued that unstable patients had balance impairment not related to loss of proprioception but rather to foot plantar-flexor weakness. The observed correlation between distal muscle strength and postural control in quiet standing of CMT patients was, however, not found in the works of Nardone et al. [13] and Van der Lindeen et al. [17]. This discrepancy can be explained by the different characteristics of the patient samples. In the study of Nardone et al, the CMT1A patients who were as stable in quiet standing as the controls, had only a slight plantar-flexor deficit, while those less stable had a more evident plantar-flexor deficit [13]. The authors found no correlation between deficits and stability, possibly because the more severely affected group was formed by 4 of the 15 subjects. With regard to the Van der Linden study, it was hypothesized that the correlation between leg muscle strength and postural sway was masked by the relatively low variability among their group of mildly affected subjects, as recognized by the authors [17]. Further investigation is needed to verify this hypothesis.

During the postural stabilization phase the role of large sensory fibres becomes apparent. These fibres are activated by dynamic stimuli, such as the flexion-extension movements of joints that occur as the subject rises from the chair. During the stabilization phase these allow the joints to reach a position of equilibrium. This is reflected in the significant correlation between vibration sense score and the global index of stabilization performance (*I*), indicating that subjects with greater large sensory nerve fibre damage have less ability to stabilize. The strength of this correlation was low, maybe because the vibration sense score is only a 5 level ordinal while parameter *I* is a continuous variable. This result is in accordance with the hypothesis of Nardone et al. [13], who suggest that these fibres have a role in the control synergies during the dynamic phase (that in this paradigm occurs during the stabilization phase) rather than in a static condition like quiet standing where the subjects have already reduced the initial instability rate *Y*_0_ to the final value *Y*_inf_. Thus it appears that not only sensory information, but also distal muscle strength influences postural stabilization. CMT1A subjects with weak distal muscle had greater difficulty in finding a stable condition (high values of *I*), and this is because, in order to reduce the initial instability rate and to stabilize joints, a sufficient level of muscle strength is needed. This is further confirmed by the relevant correlation found between parameter *I* and leg strength score, MRC_APF_ and MRC_ADF_.

The study of the STS task was a useful way to investigate the postural skills of subjects affected by CMT1A in static and dynamic conditions, and provided a more detailed insight into balance impairment than standard posturography during quiet standing. Parameter *I*, related to disease severity and was useful in understanding if the CMT subjects’ skills should be considered within the range of normality or outside of it.

## 5 Conclusions

Distal muscle weakness is an important factor that has a negative influence on both postural stabilization and quiet standing after a STS task. For this reason muscle weakness should be considered in studies on postural control in CMT1A subjects. The difficulty in maintaining erect posture appears to be mainly associated with muscle weakness, especially that of the plantar-flexors, rather than to damage of the proprioceptive system. The poor performance shown by CMT1A subjects in the stabilization phase would most likely be associated with both residual muscle strength and impaired proprioceptive feedback.

## Conflict of interest

None of the authors report a conflict of interest.
